# Backfilling behavior of a mixed aggregate based on construction waste and ultrafine tailings

**DOI:** 10.1371/journal.pone.0179872

**Published:** 2017-06-29

**Authors:** Qiusong Chen, Qinli Zhang, Chongchun Xiao, Xin Chen

**Affiliations:** 1 School of Resources and Safety Engineering, Central South University, Changsha, People’s Republic of China; 2 School of Civil and Resource Engineering, The University of Western Australia, Crawley, WA, Australia; 3 Key Laboratory of Mineral Resources Exploitation and Hazard Control for Deep Metal Mines, Changsha, People’s Republic of China; East China Normal University, CHINA

## Abstract

To study the possibility of utilizing mixed construction waste and ultrafine tailings (CW&UT) as a backfilling aggregate that can be placed underground in a mine, physicochemical evaluation, proportioning strength tests, and pumpability experiments were conducted. It was revealed that mixed CW&UT can be used as a backfilling aggregate due to the complementarities of their physicochemical properties. In addition, as the results of the proportioning strength tests show, the compressive strength of a cemented CW&UT backfilling specimen cured for 28 days, with a mass fraction of 72–74%, a cement-sand ratio of 1:12, and a CW proportion of 30%, is higher than 1.0 MPa, which meets the safety requirements and economic consideration of backfilling technology in many underground metal mines, and can also be enhanced with an increase in the cement-sand ratio. The results of the pumpability experiments show that cemented backfilling slurry based on CW&UT can be transported to the stope underground with a common filling pump, with a 16.6 MPa maximum pressure, with the condition that the time of emergency shut-down is less than approximately 20 min. All in all, the research to utilize mixed CW&UT as a backfilling aggregate can not only provide a way to dispose of CW&UT but also will bring large economic benefits and can provide constructive guidance for environmental protection.

## Introduction

Construction waste (CW) represents over one-third of the total solid waste in the world [1], as a result of city modernization and development. Since the improper disposal of CW in the environment causes several problems, such as floods, the proliferation of vectors harmful to human health, the occupation of land and the degradation of urban landscapes [2, 3], in recent years researchers have focused on the possible recycling of CW, such as the most common utilizations as recycled aggregate concrete and reclaimed asphalt pavement, and have achieved successful results with regards to durability and strength [4, 5]. However, compared with natural aggregate, the cost of CW is much higher due to the strict requirements of the construction industry, and the stability also needs to be verified by more industrial applications. Considering that backfilling mining technology plays a significant role in the protection of the environment during the recycling of industrial waste, such as tailings, slag, fly ash and ardealite [6–8], and that the requirements of strength and stability of cemented paste backfill (CPB) are relatively low, backfilling CW into the stope underground in a mine may be a good way to minimize those problems. However, the particles of CW are too coarse to be used alone as backfilling aggregate [9], even after being crushed, and the chemical factor may also have some effects. Hence, a large amount of cement is required to improve the strength and flowability of a CPB based on CW, which greatly increases the cost of backfilling. Therefore, mixing with a widely accessible, low-cost material may be an effective way to address these issues with CW, and the best option for this would also be an industrial waste.

Tailings are an industrial waste generated during mineral processing. With the ever-growing demand for mineral resources, an alarmingly large amount of tailings (approximately 1.2 billion tons per year in China) that require management has been produced [10, 11]. In this case, the utilization of tailings as a backfilling aggregate transported to the stope underground becomes a normal management strategy, owing to such advantages as less surface discharge of solid waste and excellent underground pressure control. However, with the improvement of mineral processing technology and equipment, the crude ore is now crushed to sub-micrometer particles in order to improve the recovery rate of mineral commodities from ore [12, 13], which also makes the tailings ultrafine. Unfortunately, the ultrafine tailings (UT), with an average grain diameter less than 0.03 mm, are not suitable as backfilling aggregate due to the weaknesses in strength of the mixed slurry [14, 15]. Fittingly, as mentioned in the previous paragraph, the particles of CW are coarse, which makes it possible to use CW as a backfilling aggregate after mixing with UT. In addition, the ultrafine particles of UT has a potential advantage in ameliorating the flowability of backfill [16], thus, if a mixture of construction waste and ultrafine tailings (CW&UT) can be utilized as a backfilling aggregate, it would offer a solution to certain environmental and industrial problems.

Therefore, the present paper focuses on the backfilling behavior of a mixed aggregate based on CW&UT to research the possibility of utilizing a mixed CW&UT as a backfilling aggregate. First, the physicochemical properties were evaluated to analyze the backfilling characteristics of mixed CW&UT from the material composition; then, proportioning strength tests were conducted to study the strengths of cemented CW&UT backfilling specimens with different mixing ratios, and the micro mechanisms of specimens were also analyzed; finally, pumpability experiments were carried out to study the flowability of the CPB and the reliability of transporting it to the stope underground.

## Materials and method

### Evaluation of materials

#### Materials preparation

**Fig 1:**
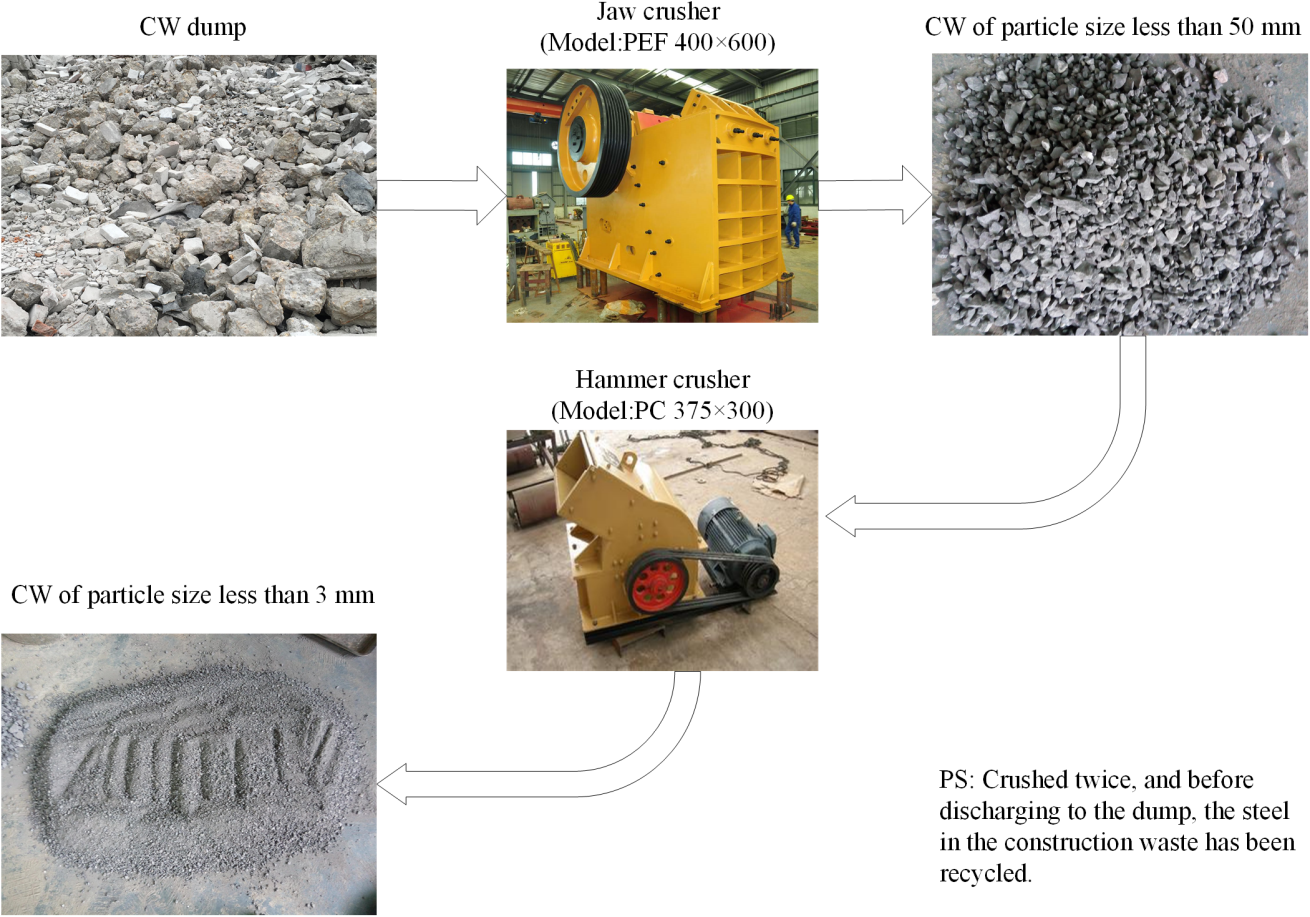
The crushing progress of CW samples.

(1) The CW utilized in this study was collected from a construction waste dump belonging to Yinpenling Community, in Changsha, Hunan Province, China, which is the closest to laboratory. The permission was granted by Yinpenlin community committee. As shown in Fig 1, the CW samples were crushed and then ground with a jaw crusher (PEF 400×600, Zhongyang located in Zhengzhou, China) and a hammer crusher (PC 375×300, Zhongyang located in Zhengzhou, China), respectively, to a particle size of less than 3 mm, to make utilization as a backfilling aggregate possible. Of course, before being discharged into the dump, the steel in the CW had been recycled, ensuring the reliability and safety of further grinding treatment.

(2) UT—The tailings utilized in this study were a type of ultrafine lead-zinc tailings, which was received from the Baoshan Lead-zinc Mine (BLM), located in Hunan Province of China.

#### Measurement of physicochemical properties

The CW and UT samples were prepared and then tested at the School of Resources and Safety Engineering, Central South University, China. To accurately measure the physical properties and chemical composition of the CW and UT, the oxides covering the surface of the samples were removed via a sand washing machine before measurement [17, 18]. The methods of measuring physical and chemical properties are as following.

1. The Sieving method and hydrometer method were used in combination to analyze the particle size of materials. Among them, sieving method was utilized to test the particles coarser than 0.075 mm, and the hygrometer was suitable for the particles finer than 0.075 mm. Firstly, the dried material of 500 g was passed the sieves with aperture of 2.0 mm, 0.5 mm, 0.25 mm, and 0.075 mm respectively, by which the particle size distribution lager than 0.075 mm was calculated. Next, 50 g material selected from the remaining sieve that finer than 0.075 mm was mixed with water in a 1000 mL measuring cylinder. This cylinder was then placed on a desk and the particles settled by gravity. After that, the particle size distribution finer than 0.075 mm was also determined through detecting the density variation of solution with time, as described in literature [19].Chemical component
2. As for backfilling technology, SiO_2_, CaO, Al_2_O_3_, Mg, etc. are the main chemical compositions of materials. The chemical composition of CW and UT was determined by using the method of X-ray fluorescence [20].

### Proportioning strength test

The proportioning strength test was conducted to determine the optimal proportion parameters, such as mass fraction, cement-sand ratio and CW proportion, which are the most important factor in backfilling system design [21], and this test is also an effective primary way to confirm whether or not CW&UT can be used as a backfilling aggregate.

#### Experimental program

According to the experiences about proportioning strength of backfill in some mines, the cement-sand ratio was designed preliminarily 1:4, 1:8 and 1:12 in consideration of the cost. Also, the mass fraction of backfill was determined 70–74%, through some simple stirring tests evaluating its flowability qualitatively before experiment [21]. Additionally, the CW proportion was designed 0%, 10%, 20%, 30%, 40%, and 50% according to some other similar materials [16]. The main purpose of this study is to explore the possibility of utilizing mixed CW&UT as a backfilling aggregate, so the simple analysis by control variable method was selected in this research, as following:

1. The cement-sand ratio was kept 1:8, the common ratio used in backfilling technology, to study the compressive strengths of specimens cured for 28 days with the CW proportion and mass fraction were changed. The optimum CW proportion then can be determined approximately;
2. Based on the optimum CW proportion, the mass fraction was kept 72% to explore the compressive strength of specimens cured for 3, 7 and 28 days with a variable cement-sand ratio. This is to select the cement-sand ratio to satisfy the common strength requirement in a backfilling mine, which can also confirm the feasibility of utilizing mixed CW&UT as a backfilling aggregate.

#### Methods of proportioning strength test

According to the experimental program described above, the quantities of the various materials were calculated and then mixed uniformly in a mixer (JJ-5, Hongda, Hebei Province of China). After being stirred for more than 5 min, the uniform backfill slurry was poured into a standard tri-unit mould 7.07 cm × 7.07 cm × 7.07 cm, and after a rest period, 9 cubic specimens were formed for each mix. In addition, the specimens were coded and placed in a curing box (style:YH-40B, Qingda, Tianjing of China) with temperature of 25°C and relative humidity of 90%. Finally, the unconfined compressive strength tests were performed on specimens cured for 3, 7, and 28 days using a WDW-2000 rigid hydraulic pressure servo machine.

### Mechanism of cemented backfilling

Scanning electron microscopy [22] and energy dispersive spectroscopy (SEM-EDS) tests [23] were conducted to analyze the microstructure of the specimens and further explore the utilization of mixed CW&UT as an aggregate.

The fractured samples obtained from strength tests were used in SEM study. The samples were cut into pieces with a size of 0.5 cm×0.5 cm, then dried in an oven at a temperature of 50°C and treated with alcohol to prevent further hydration ahead of SEM analysis. After that, a sample piece was placed on the sample table of SEM apparatus for test. Also, the EDS was analyzed by an energy spectrometer connected to SEM.

### Pumpability experiment

Proportioning strength tests studied the possibility of utilizing mixed CW&UT as a backfilling aggregate based on strength; however, it is also a key issue to be able to transport the CPB into the stope underground in a mine, which is called pumpability. As we know, the pumpability of the CPB refers to the flowability, plasticity, and stability of the backfill material during the pipeline transportation [24]; so, theoretical analysis alone is inadequate to perform the pumpability analysis, as it will cause great errors. Therefore, the circular pipe test needed to be conducted to evaluate the pumpability of the CPB based on CW&UT.

**Fig 2:**
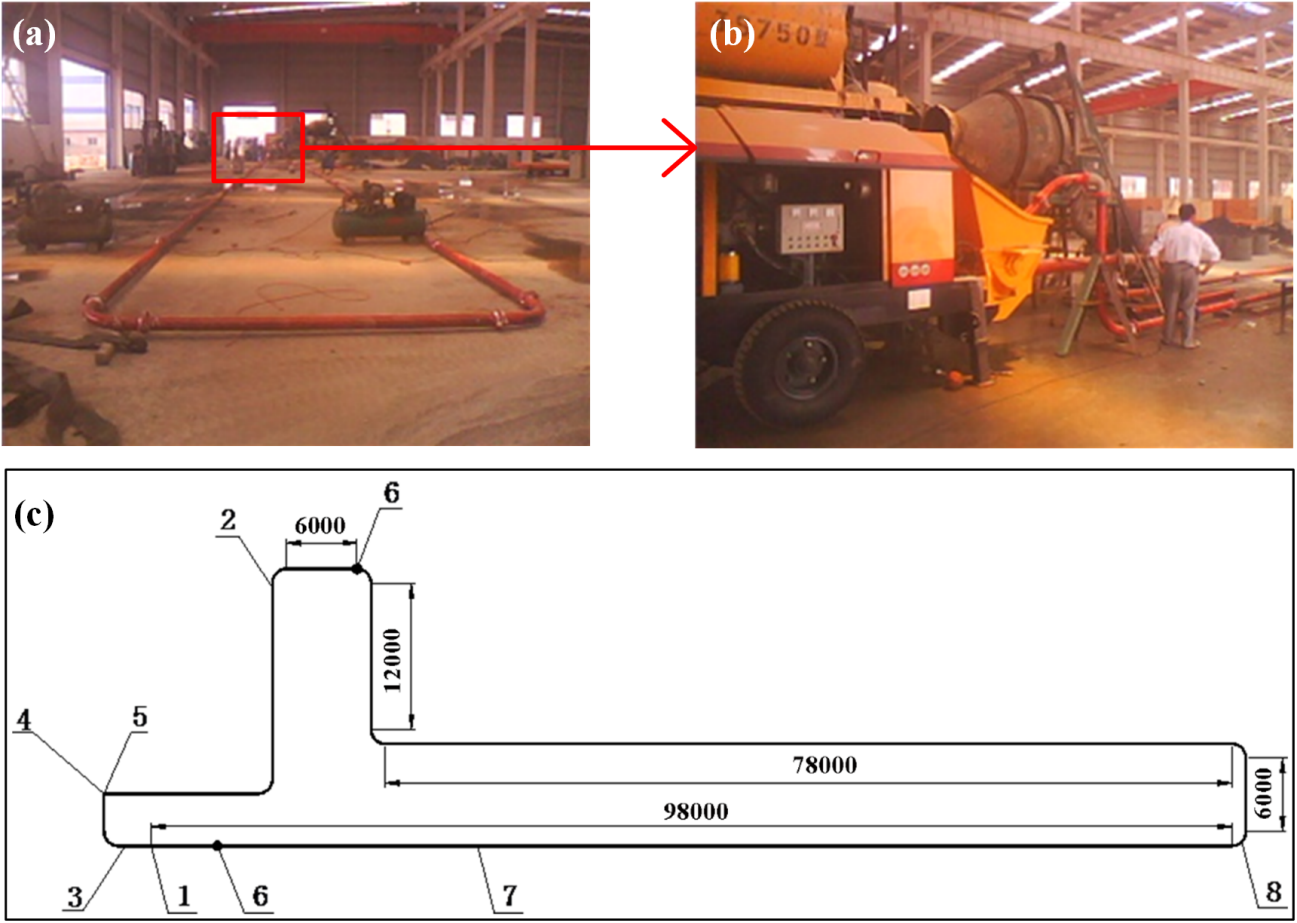
Pipe layout of the circular pipe test. (a) and (b) Site photos (c) The illustrative diagram: 1—*p*_1_, inlet pressure; 2—*p*_2_, outlet pressure; 3—velocity determination instrument; 4—HBT80/21-220S; 5—blender; 6—test position; 7—seamless steel pipe; and 8—bend.

According to the experiences of pump backfilling systems and crafts [25, 26], seamless steel pipe, with an inner diameter of 100 mm and a thickness of 7 mm, and one type of piston backfilling pump, namely, the HGBS 80.16.220 (maximum outlet pressure is 16.6 MPa, the most common one used in China), were preliminarily chosen for the circular pipes. Some other equipment, such as one type of velocity determination instrument, two pressure sensors and numerous bends with a radius of curvature of 600 mm and 90 degree right-angles, were included as well. Pipes were connected by fast joins, and the exit of the backfilling pump was joined with reducer pipes (thickness of 7 mm and length of 400 mm). The pipeline was laid horizontally, with a total length of approximately 220 m (the distance of the measuring point is 200 m), including the length of the horizontal pipeline, reducer pipes and bends. The pipe layout of the circular pipe test is shown in Fig 2. Slurry preparation was performed using three sets of 0.75 m^3^ concrete mixers with artificial feeding mechanisms. To ensure the backfilling slurry was uniform and qualified, the mixing time needed to be extended to 5 to 6 minutes.

## Results and discussion

### Evaluation of physicochemical properties

The chemical composition and particle size distribution are listed in Tables 1 and 2, respectively.

**Table 1:** Main chemical composition of the backfilling materials.

Material	Chemical composition, %					
	Fe_2_O_3_	Al_2_O_3_	SiO_2_	CaO	MgO	Others
CW	2.19	15.54	37.45	17.57	0.88	26.37
UT	3.08	4.13	19.86	38.33	9.42	25.18

**Table 2:** Particle size distributions of the backfilling materials.

Particle size range (size, mm)	Distribution (mass fraction, %)	
	CW	UT
3.0~2.0	35.0	-
2.0~0.5	25.0	3.6
0.5~0.25	22.5	8.6
0.25~0.075	8.6	7.5
0.075~0.05	3.0	10.1
0.05~0.005	3.9	59.2
<0.005	2.0	11.0

The following conclusions are suggested by the results of the physicochemical evaluation (Tables 1 and 2):

1. Al_2_O_3_ and SiO_2_ account for 15.54% and 37.45% of the contents of the CW, respectively, as shown in Table 1, which is beneficial to the strength of the backfilling body. However, MgO accounts for approximately 9.42% of the UT, and its corrosiveness may affect the strength of the backfilling body [27, 28] and may make it unsuitable as a separate aggregate. Fortunately, in both the CW and UT, CaO content is up to 17.57% and 38.33%, respectively, which could promote the formation of ettringite during the hydration reaction. This would increase the flowability and strength of the cement slurry [29].
2. CW is coarse, with particles larger than 0.5 mm accounting for 60% of the mass, and the percentage of particles smaller than 0.075 mm accounting for only 8.9%. It is well known that large particles help reinforce the strength of a backfilling body [30], and the loss of small particles leads to segregation phenomena, bad flow ability (blockage), and wear in pipelines [7]. However, the UT are fine, with particles smaller than 0.075 mm accounting for 80.3% of the mass. This will hinder dewatering of the backfilling body and reduce its strength. However, fine particles cause less wear to pipelines and contribute to the pipeline transportation of backfilling slurry [31].
3. Overall, CW and UT are not ideal backfilling aggregates, when considered separately. However, the complementarities of their physicochemical properties suggest that mixed CW&UT may be used as a backfilling aggregate.

### Proportioning strength test

**Fig 3:**
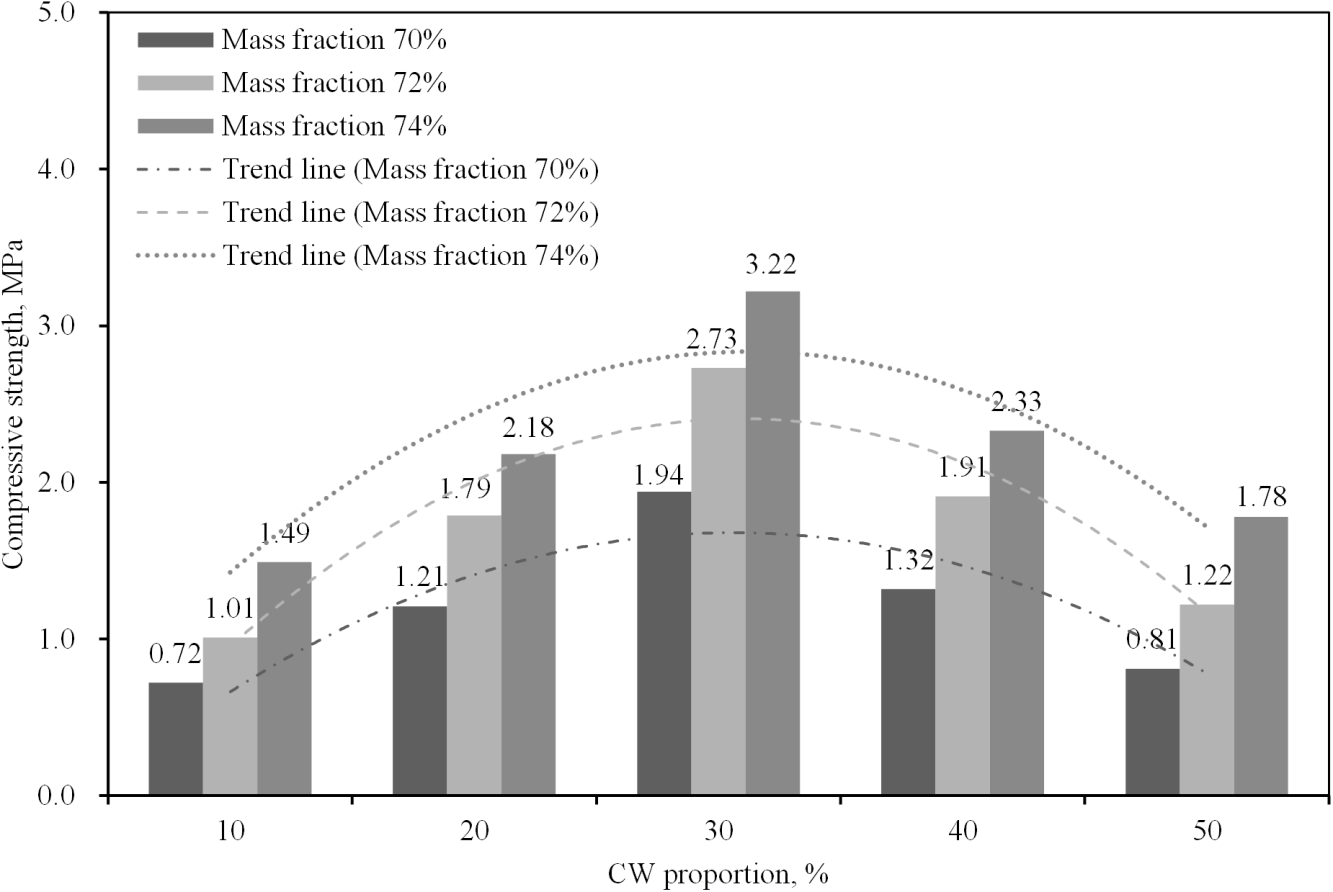
Compressive strengths of specimens cured for 28 days, with a cement-sand ratio of 1:8, when the mass fraction and construction waste proportion are changed.

**Fig 4:**
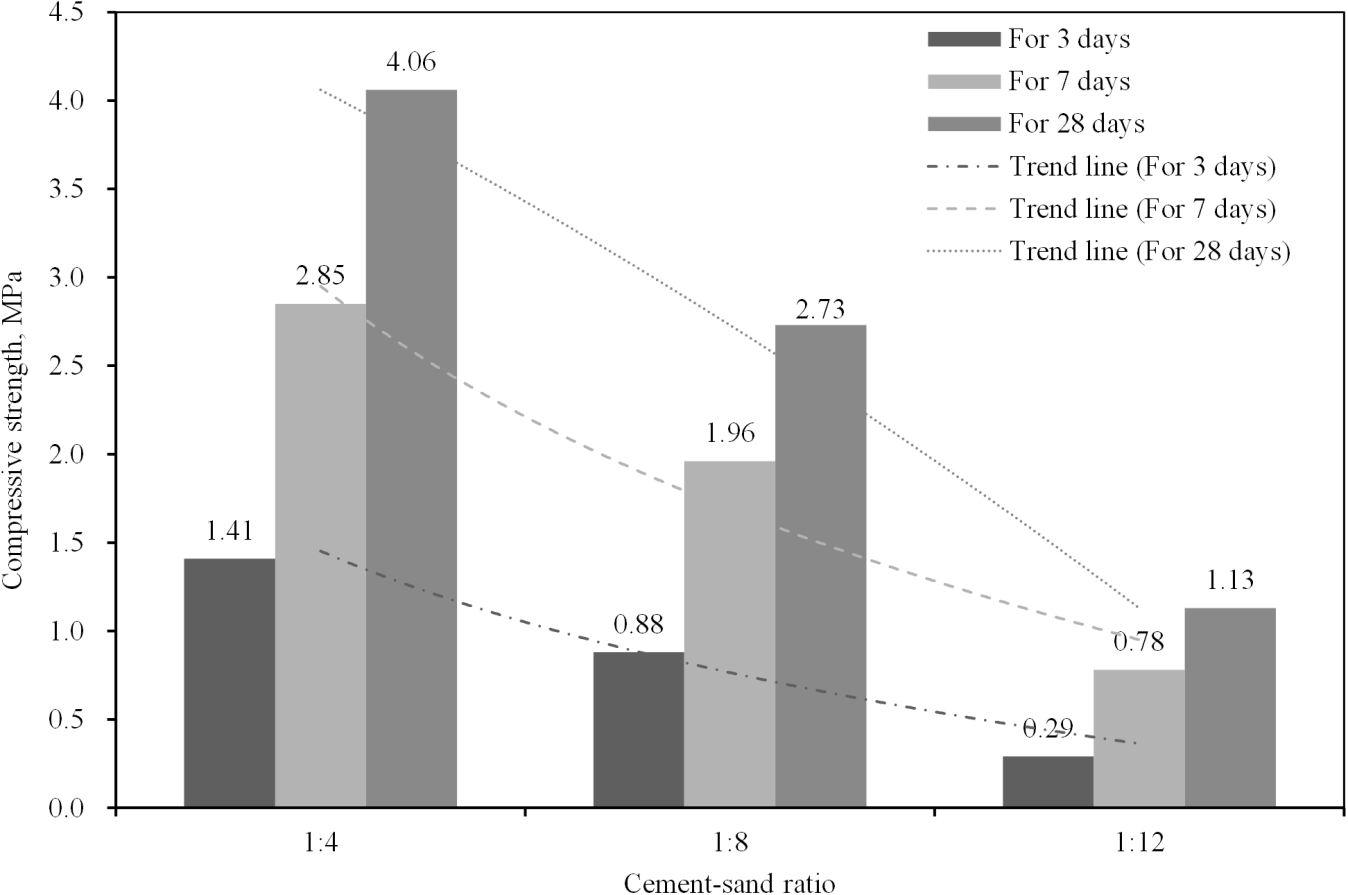
Compressive strength of specimens cured for different numbers of days with a variable cement-sand ratio and the condition that the construction proportion is 30% and the mass fraction is 72%.

**Fig 5:**
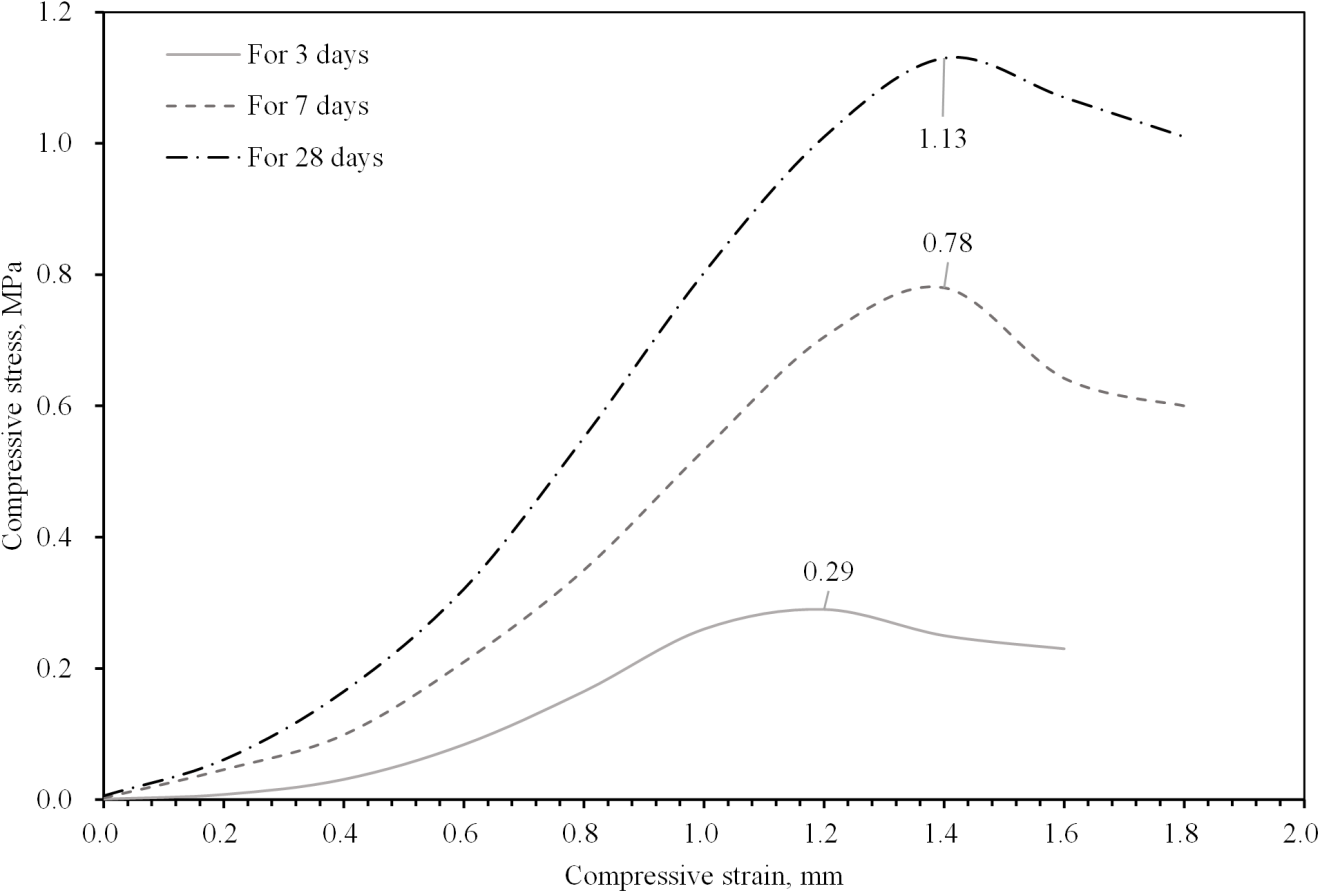
Typical stress–strain curves of specimens for different mass fractions, with the condition that the cement-sand ratio is 1:12 and the construction proportion is 30%.

The results of the proportioning strength tests are given in Figs 3–5, from which the following conclusions can be suggested:

1. Fig 3 shows the compressive strength of the specimens when the mass fraction and CW proportion are changed. As expected, the compressive strength increased as the mass fraction increased. Also, the strength presented a tendency of rising up at the beginning and declining later with CW proportion raised. As shown in the trend line of strength with construction waste proportion in Fig 3, the optimal proportion of construction waste is approximately 30%, at which the strength of the backfilling body was maximized.
2. Fig 4 shows the compressive strength of specimens cured for different numbers of days with a variable cement-sand ratio. The compressive strength clearly increased as the number of curing days increased because of the complex physicochemical effects of the hydration reaction. In field applications, the selection of the curing period depends on the strength requirements. The amount of binding material determined the strength of the backfilling body, when the other factors were left unchanged. The trend line shows the strength at different cement-sand ratios. It can be seen that the compressive strength of the backfilling body increased significantly as an increasing amount of binding material was added. However, it is necessary to reduce the amount of cement as much as possible since a higher proportion of cement increases the operating cost directly.
3. Fig 5 presents the compressive stress-strain curves, from which it can be seen that the backfilling specimens exhibited high elasticity and plasticity, allowing them to efficiently bear the weight of the stope after breakage. This also suggests that mixed CW&UT can function as a backfilling aggregate.
4. Finally, to meet safety requirements and economic considerations, the compressive strength of backfilling bodies cured for 7 days and 28 days should not be less than 0.3 and 1.0 MPa, respectively, in a mine employing the sublevel open stopping method with delayed backfill. This suggests the use of slurry with a mass fraction of 72–74%, a cement-sand ratio of 1:12, and a construction waste proportion of 30%. Of course, this is just a preliminary result based on the materials selected from Changsha and BLM, the accurate proportion should be determined by more experiments in different mines.

### Mechanism of cemented backfilling

**Fig 6:**
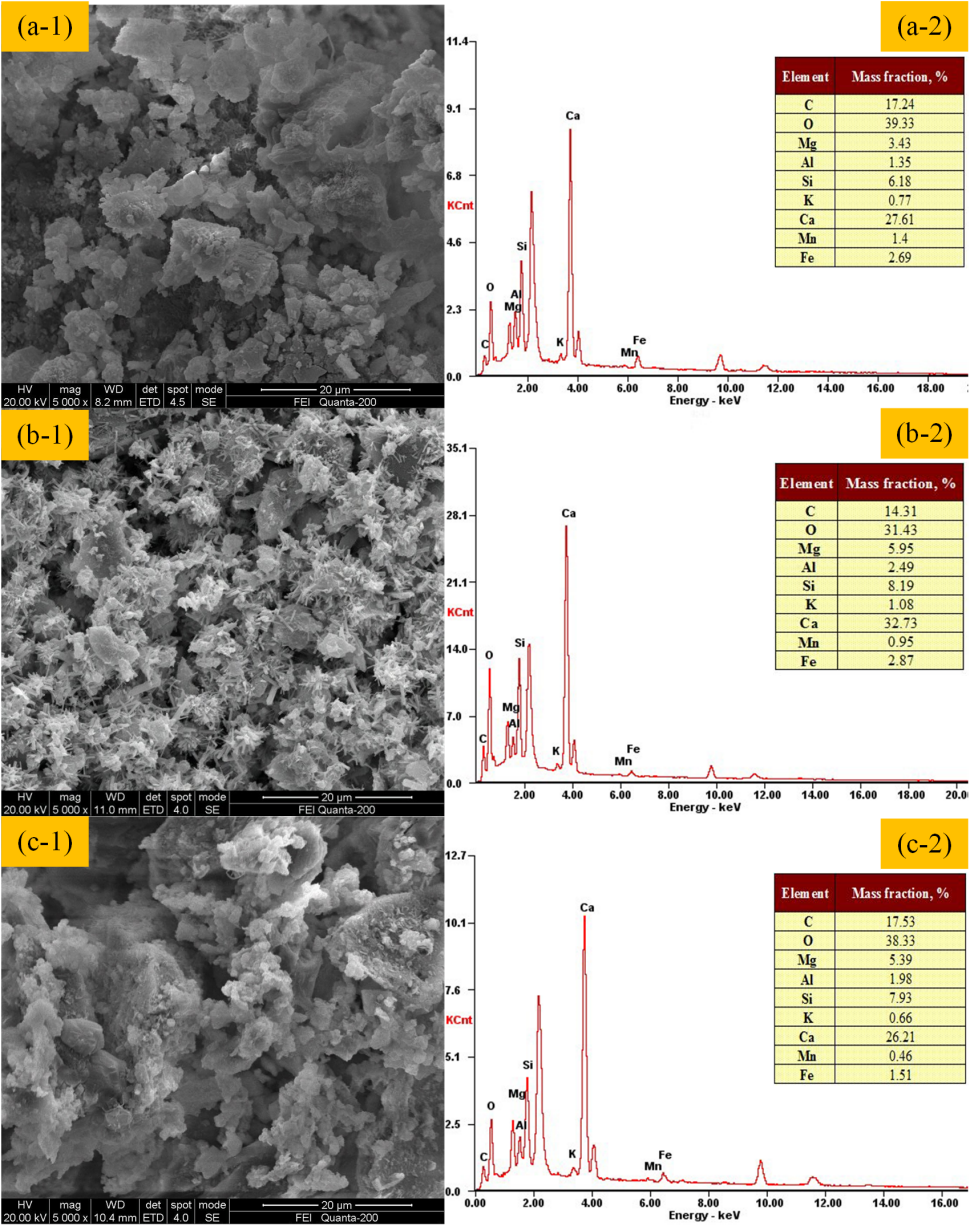
The SEM analysis of specimens cured for 28 days with the condition of a mass fraction of 74%, cement-sand ratio of 1:12, when the CW proportion is 10% (a-1), 30% (b-1), and 50% (c-1). Additionally, (a-2), (b-2), and (c-2) presents the element composition analysis by EDS correspondingly.

Fig 6 shows the SEM-EDS analysis of specimens cured for 28 days, with the condition that the mass fraction is 74% and the cement-sand ratio is 1:12, while the CW proportion is 10% (S1), 30% (S2), and 50% (S3), respectively.

As shown in Fig 6, the hydration of specimen of S2 is better than the others. It is obvious that a lot of hydration products, including spherical/needlelike C-S-H (3CaO·2SiO_2_·3H_2_O), C-H [Ca(OH)_2_] in a shape of six square plates, and rod-like AFt (3CaO·Al_2_O_3_·3CaSO_4_·32H_2_O) were produced in S2 specimen [32], while, just a little C-S-H or C-H were generated in specimens S1 and S3. Also, the hydration products shown in Fig 6B-1 wrapped the backfilling aggregate densely and uniformly.

As analyzed before, Al_2_O_3_ and SiO_2_ account for 15.54% and 37.45% in CW respectively, while they are only 4.13% and 19.86% in UT. These compositions that help to improve the effect of hydration raise with the increase of CW proportion. However, the CaO, also an important composition affecting the consolidation, especially the production of AFt [33], is much higher in UT than in CW. That is, CaO content drops with the increase of CW proportion. Thus, there should exist an optimum CW proportion that makes above relevant compositions reach a relative balanced state best for hydration. What is more, the particle size of UT is fine, while the CW is the opposite. Thus, the particle size distribution can be ameliorated with the increase of CW proportion until reaching an optimum proportion. It has been proven that an appropriate particle size distribution results in a higher strength and flowability of backfill [34]. In comprehensive consideration of the above chemical and physical factors, when the CW proportion is approximately 30%, the hydration can reach a best effect. It can also be proven through EDS results shown in Fig 6A-2, 6B-2 and 6C-2 that, the higher calcium, silicon, and aluminum contents in the S2 than the others, which means a more thorough hydration was reacted when the CW proportion is 30%.

### Pumpability experiment

**Fig 7:**
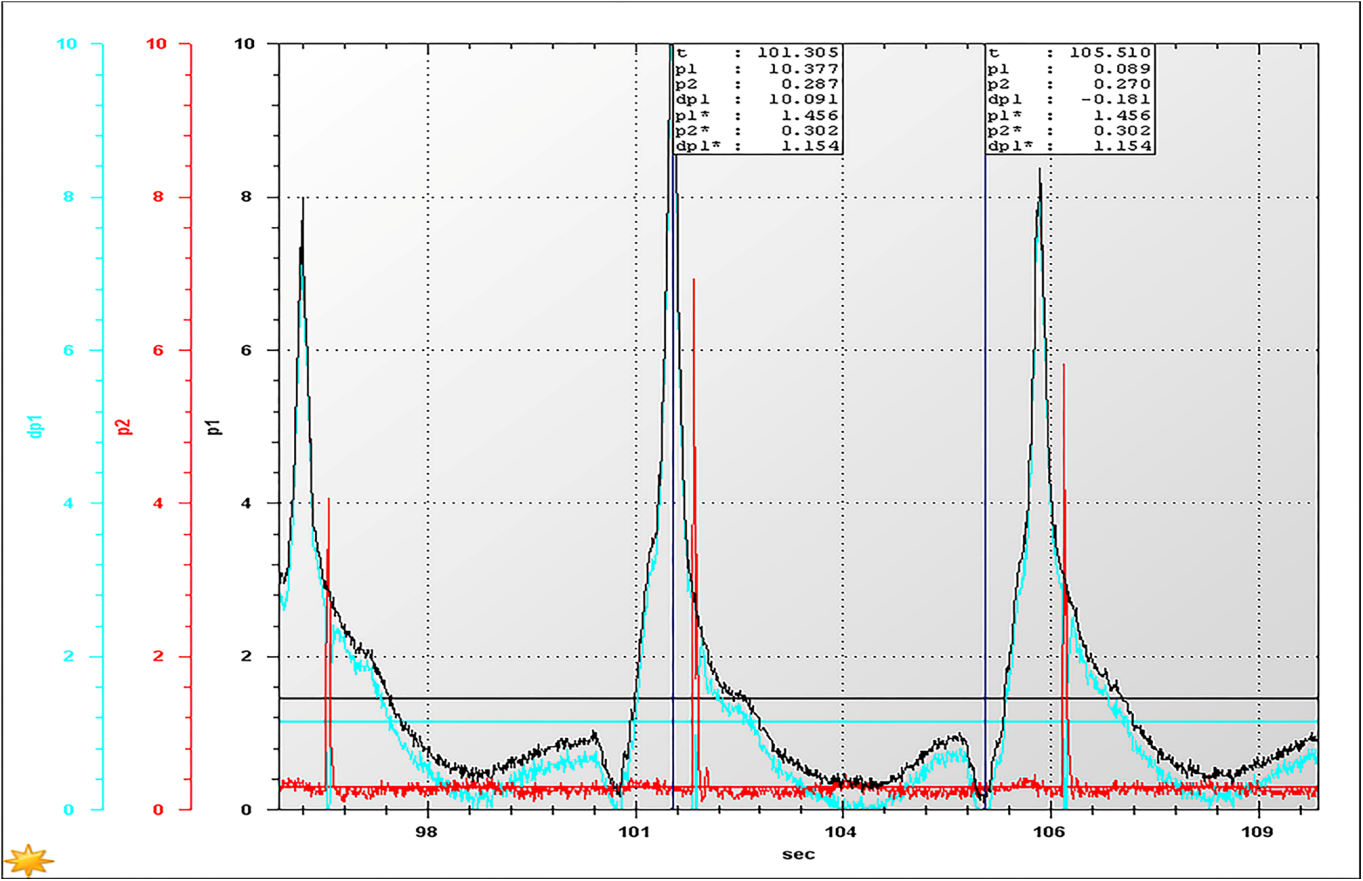
The inlet and outlet pressures at the moment of activation of the pump.

**Fig 8:**
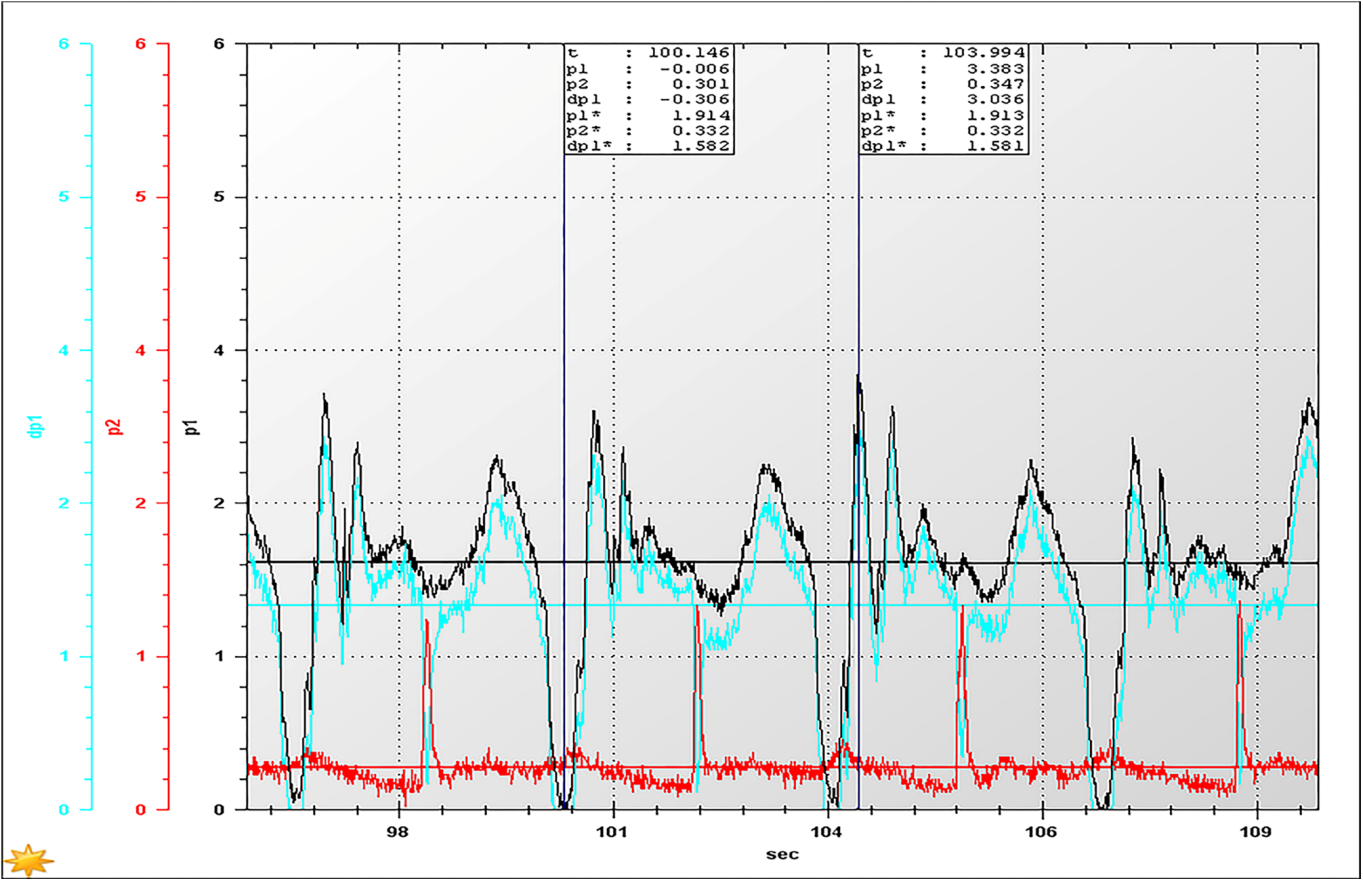
The inlet and outlet pressures at the moment of restarting the pump after 20 minutes of shut-down.

**Fig 9:**
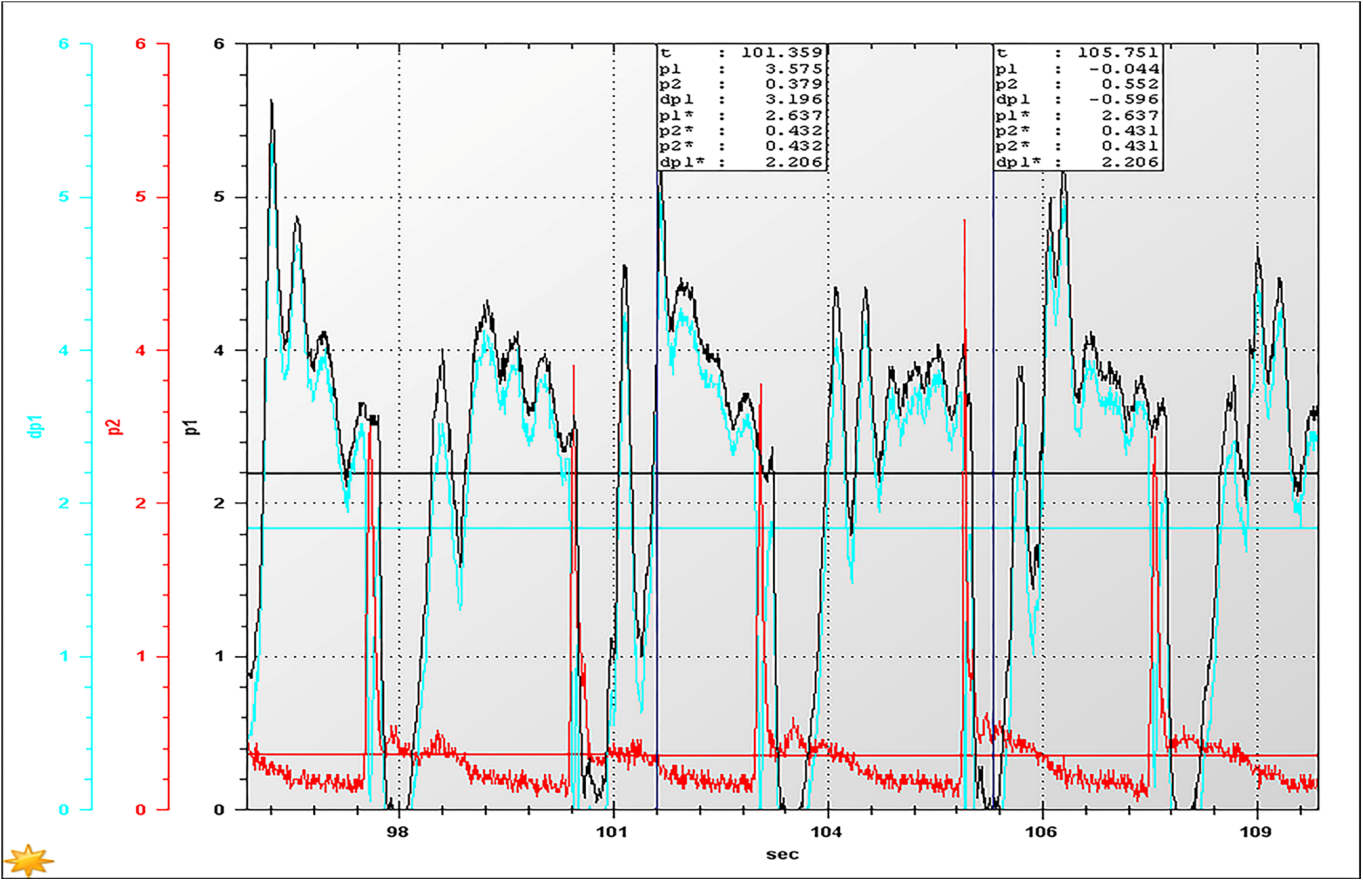
The inlet and outlet pressures at the moment of restarting the pump after 60 minutes of shut-down.

With the consideration that the lower the concentration of the slurry, the easier transportation will be, the backfill slurry with the suggested proportion (a cement-sand ratio of 1:12, and a construction waste proportion of 30%) and the higher mass fraction of 74% was tested. Accordingly, the results at the activation of the pump, restarting the pump after 20 minutes of shut-down, and restarting the pump after 60 minutes of shut-down are shown in Figs 7–9, respectively, where *p*_1_ and *p*_2_ are the values of test points 1 and 2, respectively, d*p*_1_ = *p*_1–_*p*_2_, and p* denotes the average value of *p*.

The backfilling system in BLM, with an average transmission distance of 2000 m, was used as an example to discuss the results.

1. According to Fig 7, when the pump starts, the pressure loss of the pipeline with a length of 200 meters, is approximately 1.154 MPa under the premise of a 50 m^3^/h flow rate. Therefore, the pressure loss will be presumed to be approximately 11.54 MPa when the length is 2000 meters, which suggests that the industry pump meets the requirements of a backfilling system, as the pump can provide a pressure of 16.6 MPa under low voltage.
2. As shown in Fig 8, the pressure loss (1.581 MPa/200 m) was significantly increased if the pump was restarted after 20 minutes of shut-down, indicating that the backfilling slurry has a certain degree of settlement within 20 minutes, causing an increase in pressure loss. However, the pressure loss can still be provided by the industrial backfilling pump, suggesting that the industry backfilling pump still satisfies the requirements of the stable operation of the system.
3. When restarting the pump after 60 minutes of shut-down, the pressure loss exceeds 2.0 MPa/200 m (Fig 9). Accordingly, the pressure loss of a pipeline with the length 2000 meters is reckoned to be more than 20 MPa, which fails to ensure the stable operation of the system. In this case, it is recommended to discharge the backfilling slurry residing in the pipeline with high pressure water before the pump is restarted.
4. To sum up, the HGBS 80.16.220 filling pump used for the circular pipe test is also capable of meeting the requirements of an industrial backfilling system. However, it should be noted that the maximum time required for system activation after emergency shut-down is less than 20 minutes, as the pipeline resistance largely increases because of the result of the backfilling slurry settlement in the pipeline. That is, the CPB based on CW&UT can be transported to the stope, even under the condition of a long distance of approximately 2000 m.

## Conclusions

1. CW and UT are not ideal backfilling aggregates when considered separately. However, the complementarity of their physicochemical properties allows a mixture of CW&UT to be used as a backfilling aggregate.
2. The optimal proportion of CW content in the aggregate is approximately 30%, at which level the strength of the backfilling body is maximized. We recommend the use of a slurry with a mass fraction of 72%–74%, a cement-sand ratio of 1:12, and a CW proportion of 30%. In addition, the microstructure of the specimens was analyzed to explore the reasons for utilizing the mixed CW&UT as a backfilling aggregate.
3. The pumpability of CPB based on CW&UT was tested with a circular pipe test. The results show that the HGBS 80.16.220 filling pump, with the minimum pressure 16.6 MPa, is capable of meeting the requirements of the backfilling system. Additionally, it should be noted that the maximum time required for system activation after emergency shut-down is less than approximately 20 minutes as the pipeline resistance largely increases, because of a result of the backfilling slurry settlement in the pipeline.
4. The techniques presented in this paper can not only provide a profitable and feasible backfill craft, saving a lot of cost, but also realize the reuse of industrial wastes (CW&UT). While the flowability of the CPB based on CW&UT is still a little poor, it may be a good approach to add some superplasticizer or other admixtures into the mixture, which can be studied at the next step. In addition, as the similar properties of CW, the results proposed in this study may be reproduced in a lead-zinc mine similar to BLM. However, for the complex properties of tailings in other mines, some similar proportioning strength tests based on the materials from the other mine are still required to determine the optimum proportion.
